# Supplementary material for: PIGN-Related Disease in Two Lithuanian Families: A Report of Two Novel Pathogenic Variants, Molecular and Clinical Characterisation
Source: Medicina (Kaunas). 2022 Oct 26;58(11):1526. doi: 10.3390/medicina58111526 (PMC9693321; doi:10.3390/medicina58111526)
Supplement: Supplementary file 1 [file medicina-58-01526-s001.zip › Supplementary figure 1.pdf]

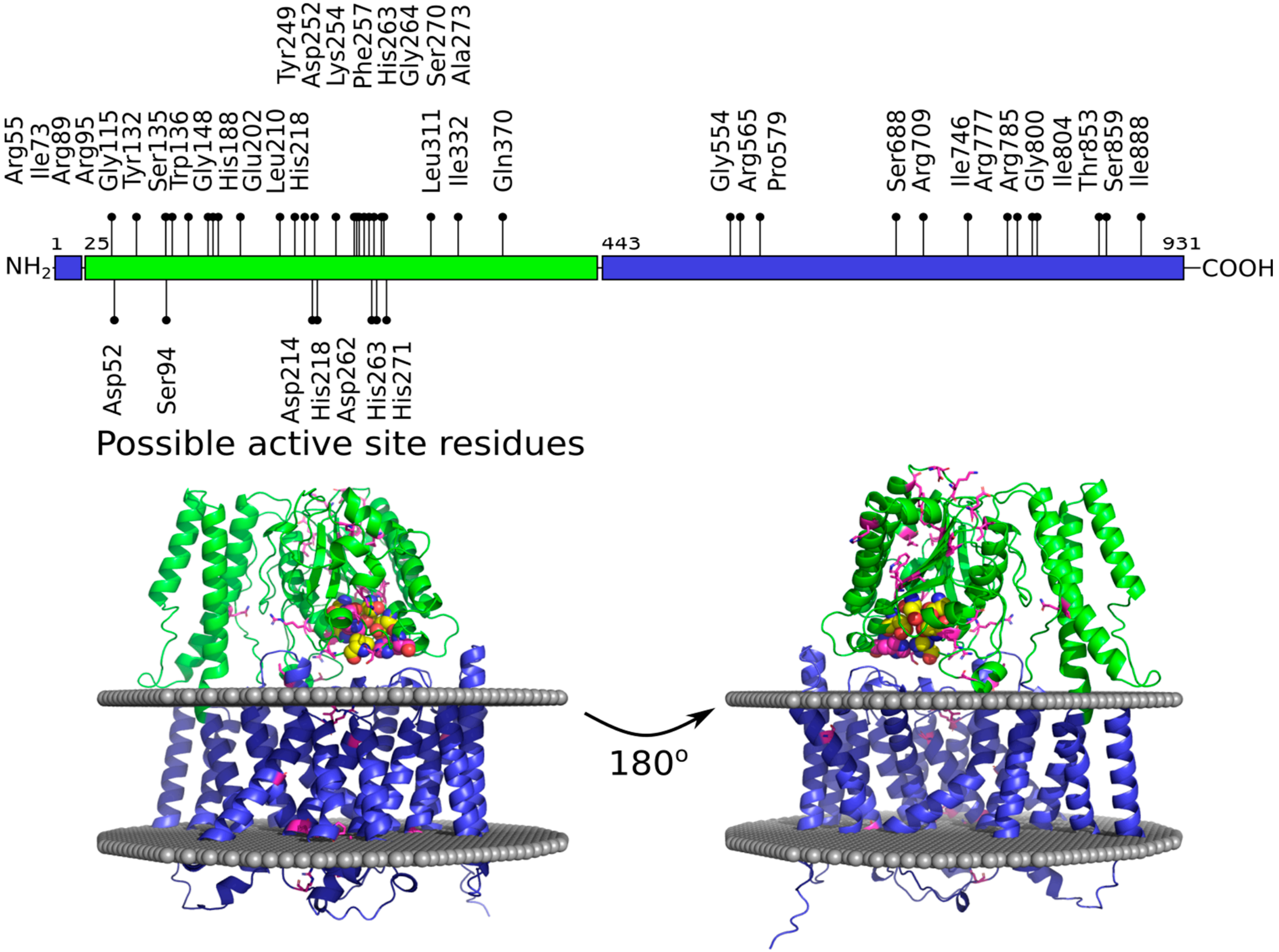

Supplementary figure 1. Currently known amino acid changing variants in the structural model of the PIGN protein. Membrane region is colored in blue, lumenal domain is green, mutated residues are colored in magenta, conserved residues that are likely to belong to enzyme active site are shown in yellow spheres
