# Supplementary material for: PIGN-Related Disease in Two Lithuanian Families: A Report of Two Novel Pathogenic Variants, Molecular and Clinical Characterisation
Source: Medicina (Kaunas). 2022 Oct 26;58(11):1526. doi: 10.3390/medicina58111526 (PMC9693321; doi:10.3390/medicina58111526)
Supplement: Supplementary file 1 [file medicina-58-01526-s001.zip › Supplementary table 2_Targeted NKS gene list.pdf]

Supplementary table 2. The list of the genes included in the Ion AmpliSeq™ IAD197386 Panel for targeted sequencing.

AFG3L2	CAD	D2HGDH	FOLR1	HTRA1	NBEA	PRICKLE1	SCN1A	SYNGAP1	NDUFS6	AP3B2
AGA	CASK	DCX	FOXRED1	HTT	OFD1	PRODH	SCN1B	SYNJ1	NFU1	TREX1
AIFM1	CASR	DDC	GABBR2	IQSEC2	OPHN1	PRRT2	SCN2A	ZEB2	PIGN	DPYS
ABAT	CDKL5	DEPDC5	GABRA1	KCNMA1	NDST1	PSAP	SCN3A	ZFYVE26	TUBB4A	FAR1
ABCD1	COX15	DHFR	GABRB2	KCNQ2	NDUFAF5	PTS	SCN8A	TBC1D24	RMND1	GFM1
ANKRD11	COX6B1	DYRK1A	GABRB3	KCNQ3	NDUFAF6	QARS	SCN9A	TBCE	RNASEH2A	GTPBP3
AP4B1	CHD2	EIF2B2	GABRG2	KCNT1	NDUFS2	QDPR	SCO1	TBL1XR1	RNASEH2C	TSC1
AP4E1	CHRNA4	EIF2B3	GALC	KDM5C	NDUFS4	PLP1	SDHAF1	TCF4	ROGDI	TSC2
AP4M1	CHRNB2	EIF2B4	GAMT	MACF1	NDUFS7	PNKP	SOX10	MFSD8	SLC25A22	MOCS1
ARG1	CLCN2	EIF2B5	GCDH	KIF1A	NDUFS8	PNPO	SPATA5	BRAT1	SLC35A2	PIGO
ARHGEF9	CLN3	ETFA	GCH1	KMT2E	NDUFV1	POLG	SPTAN1	MOCS2	SYN1	CNPY3
ARID1B	CLN6	ETFB	GNE	L2HGDH	NEDD4L	SAMHD1	VPS13A	PPT1	TPK1	UBE3A
ARSA	CNTNAP2	ETFDH	GPHN	LGI1	NEU1	SCARB2	ST3GAL3	RNASEH2B	UBE2A	ICK
ASAH1	COL4A1	DNAJC5	GRIA3	LMNB1	NHLRC1	RELN	ST3GAL5	ADAR	WDR45	PIGV
ASNS	C12orf57	FAM126A	GRIK2	KCNA1	NOTCH3	RNASET2	STAMBP	AIMP1	CDK9	KIAA2022
ASPA	CACNA1A	ECHS1	GRIN1	KCNA2	NPRL3	RNF216	WWOX	CC2D1A	ADAM22	FUT8
ASXL3	CACNA1D	ECM1	GRIN2A	KCNC1	NRXN1	SERAC1	KCNB1	CLN5	CLCN4	SMARCA2
ATIC	CACNA1E	EEF1A2	GRIN2B	KCNH1	NT5C2	SERPINI1	TAF1	CLN8	CNKSR2	SMC1A
ATP13A2	CACNA1H	EFHC1	GRN	KCNJ10	NUBPL	SLC6A8	DARS2	SLC25A1	DOCK7	SMS
ATP1A3	CACNB4	EIF2B1	HACE1	MTFMT	PLCB1	SLC9A6	ETHE1	AP4S1	HNRNPU	SNAP25
ATRX	CPT2	DNM1	HCN1	MTHFR	PCDH19	SIK1	FA2H	CHRNA2	NECAP1	
ALDH3A2	CSF1R	DNM1L	HIBCH	MTOR	PGK1	SLC12A5	MARS2	EARS2	PIGG	
ALDH4A1	CSTB	DPYD	GLB1	MAGI2	PHF6	SLC13A5	STX1B	GLRB	PIGQ	
ALDH5A1	CTC1	GFAP	GLDC	MECP2	PIGA	SLC19A3	STXBP1	GOSR2	MRPL44	
ALDH7A1	CTSD	FARS2	GNAO1	MED12	POLR3A	SLC1A2	YY1	KCTD7	SZT2	
AMACR	CTSF	FDFT1	GNB1	MED17	POLR3B	SLC2A1	SLC25A15	LRPPRC	UNC80	
AMT	CUL4B	FH	HSD17B10	MEF2C	RAB39B	SLC46A1	SUMF1	MBD5	ALG13	
BTD	CYP27A1	FLNA	HSPD1	MLC1	PPP3CA	SLC6A1	SUOX	NDUFAF3	ABCA2	
