# Supplementary material for: PIGN-Related Disease in Two Lithuanian Families: A Report of Two Novel Pathogenic Variants, Molecular and Clinical Characterisation
Source: Medicina (Kaunas). 2022 Oct 26;58(11):1526. doi: 10.3390/medicina58111526 (PMC9693321; doi:10.3390/medicina58111526)
Supplement: Supplementary file 1 [file medicina-58-01526-s001.zip › Supplementary table 3.pdf]

Supplementary table 3. The conditions for PCR amplification of *PIGN* in gDNA samples

Gene (RefSeq)	Variant	Primers (5' → 3')	Location	Annealing temperature °C	Product length
PIGN (NG_033144.1, NM_176787.5)	NM_176787.5:c.1247_1251del	**F:** CCCTGTTTGGCTCAGGGATA	Intron 13	64.6	594 bp
		**R:** CTGGCCTGCCTACTTTGCT	Intron 14		
	NM_176787.5:c.1942G>T	**F:** AGGGAGCTGTTTTTCCTGCT	Intron 19	65.0	918 bp
		**R:** CTGGGCTGCCTCTTATGGAC	Intron 20		
	NM_176787.5:c.932T>G	**F:** CTTCCATCTTTCCTTCCTCAGC	Intron 10	63.5	511 bp
		**R:** GCCACAAGAGAGATTAGCTGG	Intron 11		
	NM_176787.5:c.1674+1G>C	**F:** GCCCTTGAAACAAATCCGTTAG	Intron 17	63.0	648 bp
		**R:** TGGCCCATTTTGATGTGAAAGT	Intron 19		
