# Supplementary figures and images for: PIGN-Related Disease in Two Lithuanian Families: A Report of Two Novel Pathogenic Variants, Molecular and Clinical Characterisation
Source: Medicina (Kaunas). 2022 Oct 26;58(11):1526. doi: 10.3390/medicina58111526 (PMC9693321; doi:10.3390/medicina58111526)

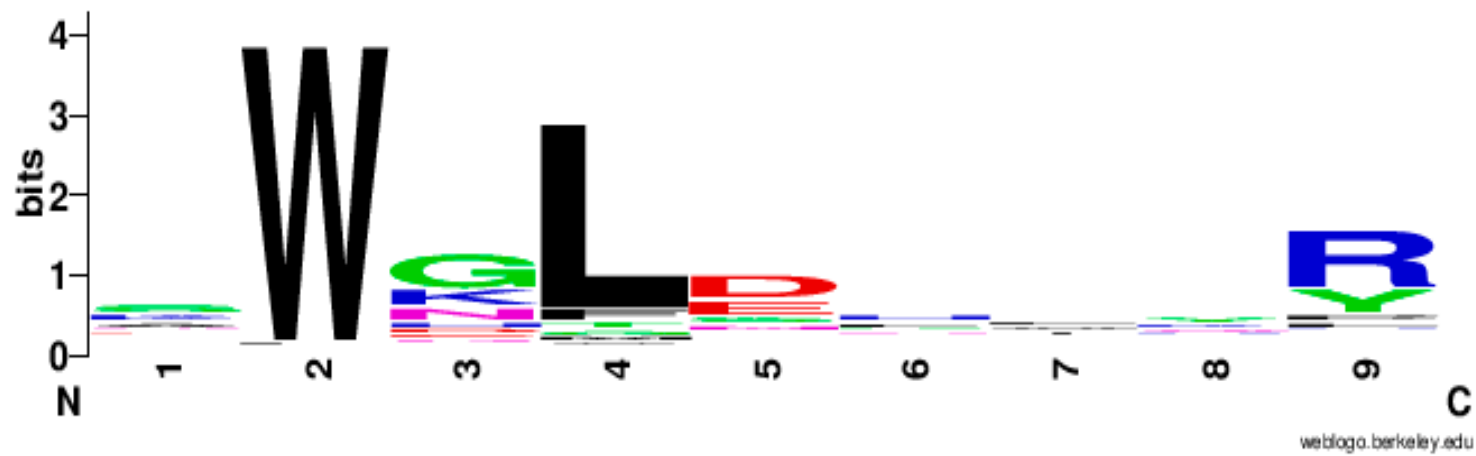

**Supplementary figure 2.** Conservation of the motif containing the Leu311 residue.

Supplement: Supplementary file 1 [file medicina-58-01526-s001.zip › Supplementary figure 2.pdf]
